# Day case endoscopic excision of an antrochoanal polyp in a paediatric patient

**DOI:** 10.1093/jscr/rjab234

**Published:** 2021-06-07

**Authors:** Manuel Bakheet, Noora Althawadi, Sultan Albinali, Mohamed Alreefy, Hiba Alreefy

**Affiliations:** Department of Otolaryngology, King Hamad University Hospital, Busaiteen, Bahrain; Department of Otolaryngology, King Hamad University Hospital, Busaiteen, Bahrain; Department of Otolaryngology, King Hamad University Hospital, Busaiteen, Bahrain; Department of Otolaryngology, King Hamad University Hospital, Busaiteen, Bahrain; Department of Otolaryngology, King Hamad University Hospital, Busaiteen, Bahrain

## Abstract

Antrochoanal polyps (ACP; also known as Killian’s polyp) are considered to be the most common type of choanal polyps, making up ~4–6% of all nasal polyps in the general population and ~33% of nasal polyps in the paediatric age group. Patient’s suffering from ACP range between the ages of 5 and 80 years. Only 4% of the patients are children aged < than 10 years old. Overall there is a male predominance making up ~64% of the total number of patients. We report a case of an antrochoanal polyp in a 9-year-old girl who presented with complaints of unilateral nasal obstruction, snoring and obstructive sleep apnea. The patient underwent a computed tomography scan and was managed endoscopically for excision of the polyp, as a day case procedure.

## INTRODUCTION

Antrochoanal polyps (ACP) are considered to be amongst the most common choanal polyps [1, 2]. They make up ~4–6% of all nasal polyps in the general population and a third of nasal polyps in the paediatric age group [3, 4]. Patient’s suffering from ACP usually range between the ages of 5 and 80 years with a mean age of 40. About only 4% of patients are younger than 10 years old [1].

## CASE REPORT

A 9-year-old girl attended the outpatient Otolaryngology clinic at our hospital with complaints of unilateral nasal obstruction, snoring and obstructive sleep apnea (OSA). Her symptoms started 5-month prior to presentation and had been progressively getting worse. The girl was noticed by her parents to have increased somnolence and reduced attention during the day. Her general practitioner offered intranasal corticosteroids but with no benefit.

**Figure 1:**
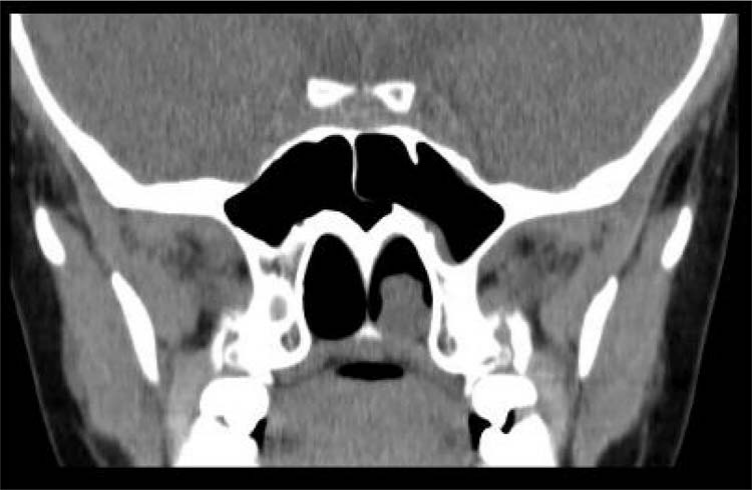
CT scan—coronal cut showing a homogenous mass occupying the inferior aspect of the left choana.

**Figure 2:**
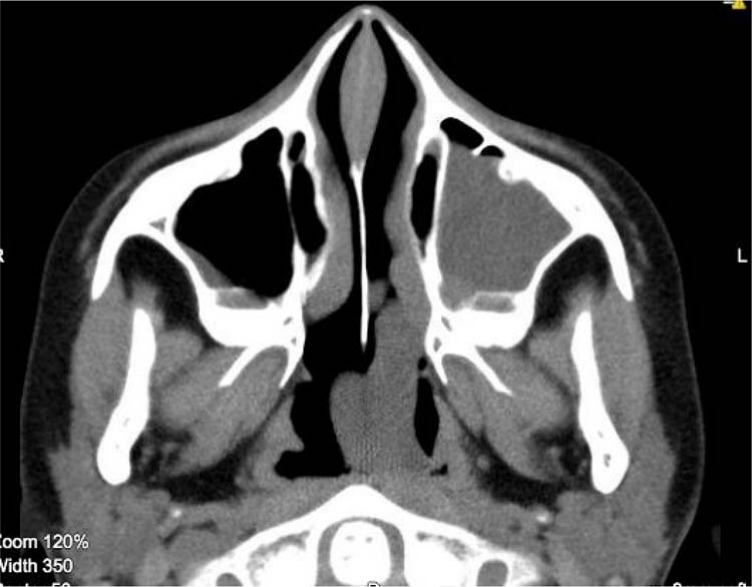
CT scan axial cut showing a large homogenous mass extending across the left nasal cavity.

Anterior rhinoscopy revealed hypertrophied inferior turbinates with allergic nasal mucosa and increased nasal secretions with no mass seen. There was no active pus discharge from the nose. On throat examination, a large red, strawberry shaped mass was seen hanging posteriorly in the oropharynx. Flexible nasal endoscopy (FNE) of the left nostril was done, revealing a large polyp extending from the middle meatus into the choana and a clear right airway. A computerized tomography (CT) scan was done (Figs 1 and 2) showing a soft tissue mass arising from the left middle meatus, extending posteriorly and reaching the left choana (Fig. 2).

An endoscopic excision of left antrochoanal polyp in the day case unit was conducted the following week.

**Figure 3:**
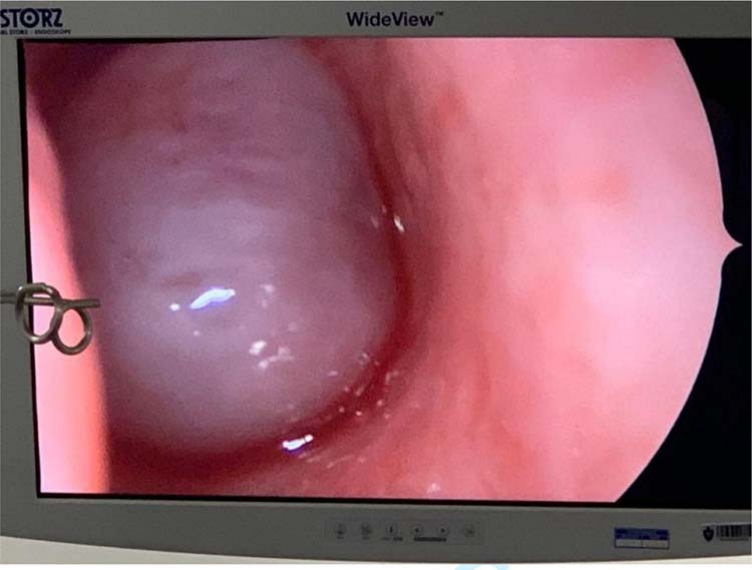
The cystic component of the ACP seen within the left nasal cavity on nasal endoscopy.

Under general anaesthesia, a large congested mass was seen extending from the left maxillary antrum into the choana (Fig. 3).

**Figure 4:**
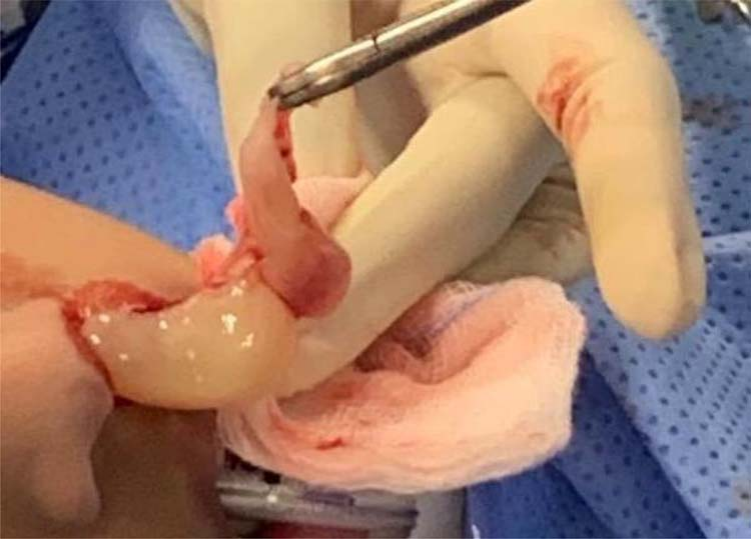
Both the cystic and the solid components of the ACP can be visualized as it has removed from left nasal cavity.

Medialization of the middle turbinate was done followed by an uncinectomy. The cystic nasal component was removed using blakesley forceps and a middle meatal antrostomy was done (Fig. 4).

**Figure 5:**
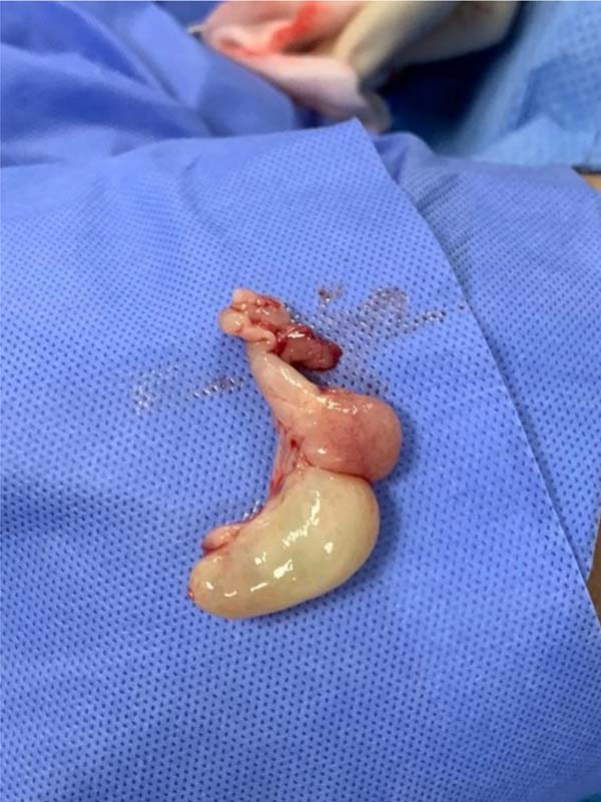
The ACP after removal from the left nasal cavity (measuring 40 × 30 × 15 mm).

The solid oral component of the antrochoanal polyp was large in size (Fig. 5), but with manipulation it was delivered transnasally. Lateralization of the middle turbinate was done and haemostasis achieved. The polyp was then sent for histopathological examination.

The patient was observed for 4-h postoperatively in the hospital with no active complaints. She was discharged on the same day on nasal decongestants, intranasal steroids and analgesia. She returned to the clinic 1-month postoperatively with significant improvement in breathing, quality of sleep and school performance. FNE showed a patent left airway with no recurrence of disease. The pathology of the polyp was reported to be of inflammatory nature suggestive of ACP.

## DISCUSSION

Nasal polyps are pedunculated, benign masses that arise secondary to chronically oedematous and inflamed nasal mucosa. They can be divided into two main types depending on their site of origin—either ethmoidal occurring in the middle meatus or choanal arising from the maxillary antrum [3, 4]. The most common site of origin for nasal polyps is the Ethmoid Sinus which are usually multiple and small in size [4]. Choanal polyps on the other hand are characteristically solitary, unilateral and larger with a pear-like appearance [1].

ACP arise from the maxillay antrum extending through the maxillary ostium, to the choana and may finally prolapse into the nasopharynx [1, 5]. These polyps are usually composed of two parts—a cystic and a solid part. The cystic antral part is usually situated at the maxillary sinus with the solid part arising from the accessory or natural ostium, extending towards the middle meatus and into the choana [1, 2, 6].

The pathophysiology of ACP has been unclear for many years with several theories hypothesized to explain its development. Amongst the most significant theories is that chronic inflammation and allergies are the main trigger. An associated inflammatory anatomical alteration at the level of the ostium and middle meatus results in a change in the pressure gradient within the maxillary sinus leading to protrusion of the polyp into the nasal cavity [1, 7].

Clinical manifestations of ACP have varied amongst populations. The most common symptom is unilateral nasal obstruction [1, 6]. Symptoms of Eustachian tube dysfunction with subsequent ear fullness, hearing loss and potentially middle ear effusion may occur [6]. Other symptoms include rhinorrhoea, purulent nasal discharge, postnasal drip as well as shortness of breath. More significant complaints include epistaxis, rhinophonia, OSA, dysphagia and weight loss [2, 3, 6].

Nasal endoscopy and radiological assessment with a CT scan are gold standard for the diagnosis of ACP [5, 7, 8]. ACP is visualised by nasal endoscopy as a smooth polypoid mass emerging from the middle meatus, extending to the choana and nasopharynx [8]. The main finding on a CT scan is a soft tissue mass that does not erode into adjacent bony structures. CT scan is also used for the staging classification of ACP [8]. Magnetic resonance imaging may be used to aid the diagnosis and exclude angiomatous polyps [8].

Diagnostic confirmation is by histopathology of the polyp, demonstrating pseudostratified ciliated epithelium which may contain oedematous and vascular stroma. It also demonstrates an infiltration of variable inflammatory cells. The scant presence of submucous glands and epithelial goblet cells is highly suggestive of choanal polyps [3, 7, 9].

The treatment of ACP is primarily surgical with functional endoscopic sinus surgery (FESS) being the treatment of choice with shorter recovery time with minimal risks, especially in children [3, 7, 8, 10]. Other surgical approaches include FESS with transcanine sinuscopy, mini Caldwell-Luc, Caldwell-Luc or simple polypectomy. These can be used either alone or in combination [11]. Complete removal of the polyp is crucial in order to reduce risk of recurrence [3, 11, 12].

## CONCLUSION

Thorough clinical assessment of patients who present with nasal obstruction can avoid unnecessary usage of medications and identifies patients who require surgical intervention. ACP can have significant impact on a child’s daily life and school performance, therefore, prompt diagnosis and treatment are required. With the drift towards endoscopic excision of ACP, surgical risks are minimised and hospital stays are shortened. Our case proves that it is safe to perform this surgery in a day case setting in carefully selected patients.

## CONFLICT OF INTEREST STATEMENT

None declared.
